# Deep learning classification of early normal-tension glaucoma and glaucoma suspects using Bruch’s membrane opening-minimum rim width and RNFL

**DOI:** 10.1038/s41598-020-76154-7

**Published:** 2020-11-04

**Authors:** Sat byul Seo, Hyun-kyung Cho

**Affiliations:** 1 Department of Mathematics Education, School of Education, Kyungnam University, Changwon, Republic of Korea; 2 Department of Ophthalmology, Gyeongsang National University Changwon Hospital, Gyeongsang National University, School of Medicine, 11 Samjeongja-ro, Seongsan-gu, Changwon, Gyeongsangnam-do 51472 Republic of Korea; 3 Institute of Health Sciences, School of Medicine, Gyeongsang National University, Jinju, Republic of Korea

**Keywords:** Optic nerve diseases, Diagnosis, Outcomes research

## Abstract

We aimed to classify early normal-tension glaucoma (NTG) and glaucoma suspect (GS) using Bruch’s membrane opening-minimum rim width (BMO-MRW), peripapillary retinal nerve fiber layer (RNFL), and the color classification of RNFL based on a deep-learning model. Discriminating early-stage glaucoma and GS is challenging and a deep-learning model may be helpful to clinicians. NTG accounts for an average 77% of open-angle glaucoma in Asians. BMO-MRW is a new structural parameter that has advantages in assessing neuroretinal rim tissue more accurately than conventional parameters. A dataset consisted of 229 eyes out of 277 GS and 168 eyes of 285 patients with early NTG. A deep-learning algorithm was developed to discriminate between GS and early NTG using a training set, and its accuracy was validated in the testing dataset using the area under the curve (AUC) of the receiver operating characteristic curve (ROC). The deep neural network model (DNN) achieved highest diagnostic performance, with an AUC of 0.966 (95%confidence interval 0.929–1.000) in classifying either GS or early NTG, while AUCs of 0.927–0.947 were obtained by other machine-learning models. The performance of the DNN model considering all three OCT-based parameters was the highest (AUC 0.966) compared to the combinations of just two parameters. As a single parameter, BMO-MRW (0.959) performed better than RNFL alone (0.914).

## Introduction

Glaucoma is caused by the injury of retinal ganglion cells (RGC) and their axons, bringing about defects in the retinal nerve fiber layer (RNFL) and the neuroretinal rim (NRR) that can result in visual field (VF) defects^1^. Detection of early structural damage is more important than detecting a functional defect in the diagnosis of early glaucoma^2,3^ because a detectable structural defect may occur ahead of visual functional loss at an individual level^4–6^. As structural change is minimal in glaucoma suspect or glaucoma of early stage, the results of different structural tests may not demonstrate consistent findings. For example, optical coherence tomography (OCT) parameters of Bruch’s membrane opening-minimum rim width (BMO-MRW) and peripapillary RNFL thickness may show discrepancies. Therefore, discriminating early-stage glaucoma from glaucoma suspect (GS) is challenging.

The clinical interpretation of abnormalities of RNFL and BMO-MRW frequently depend on the diagnostic report of color code classification. This diagnostic classification categorizes the measurement values into three classes of different color (green: within normal limits, yellow: borderline, and red: outside normal limits) based on the normative data built in the OCT device. High specificity was noticed in the diagnostic color code classification report because the first and the fifth percentile of the normative RNFL thickness/BMO-MRW data were employed in determining the abnormalities of RNFL thickness/BMO-MRW^7^. We previously reported on the discrepancy between BMO-MRW and RNFL color code classification^8^. We found that BMO-MRW may show a normal classification, whereas RNFL may show an abnormal classification in cases of large disc and myopia, which suggests the clinical usefulness of BMO-MRW in early glaucoma or glaucoma suspect when the RNFL color code classification may show false-positive findings^8^. Glaucoma of early stage is especially important since the decision to initiate lifetime treatment should be made. Moreover, an incorrect diagnosis of glaucoma may lead to unnecessary lifetime treatment.

It is more challenging to discriminate the early stage of glaucoma from glaucoma suspect or normal subjects than the advanced stage of glaucoma^9–11^. With the recent remarkable progress in the field of artificial intelligence (AI), the deep-learning method may be beneficial to aid clinicians in this situation^12–14^. However, distinguishing early glaucoma from healthy or GS is still difficult, even with deep learning, and the studies on early glaucoma are still scarce. The diagnostic performance of discriminating early glaucoma from normal controls in previous studies ranged from an area under the receiver operating characteristic curves (AUROCs) of 0.830^14^ to 0.896^12^, and in another study from 0.77 to 0.97, depending on the input maps in classifying early glaucoma from healthy/glaucoma suspects^15^. Moreover, it may be more challenging to distinguish early glaucoma from GS than from a normal control.

**Figure 1:**
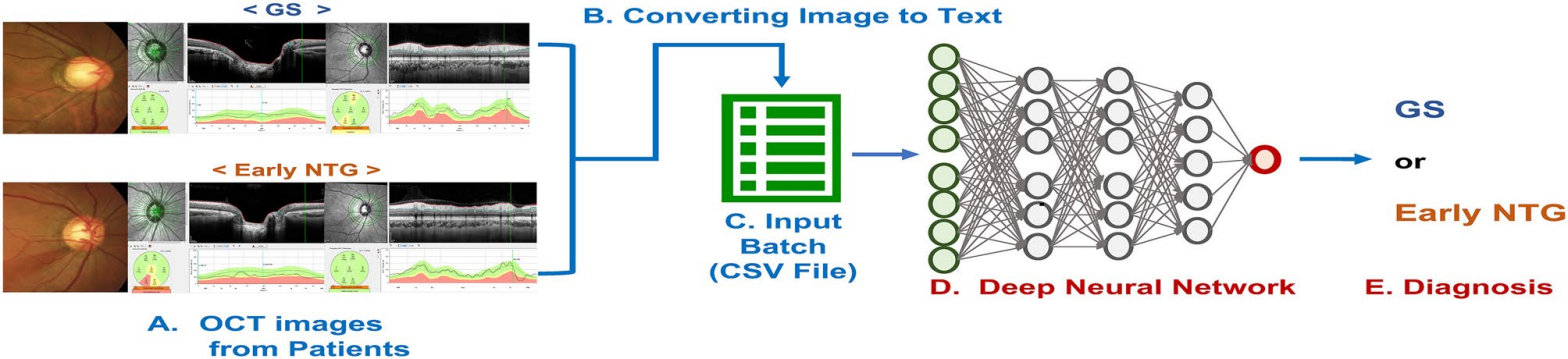
Deep neural network architecture. (**A**) OCT from patients. The upper row indicates a representative glaucoma suspect case and the lower row indicates that of early NTG. Fundus photo is shown on the left, a BMO-MRW image in the middle, and an RNFL image in the right from the same patient. Note that the color code classification of BMO-MRW and RNFL did not correspond with each other in this early stage of glaucoma or GS. (**B**) Converting image to text. Numeric values extracted from BMO-MRW and RNFL images of subjects with GS and early NTG. (**C**) Input batch. CSV file generated as input data including class (either GS or early NTG). (**D**) Deep neural network. Twenty-five parameters as input in the layer. The 1st hidden layer and the 2nd hidden layer had 10 neurons with activation functions as a rectified linear unit (ReLU), the 3rd hidden layer had five neurons with an activation function as a ReLU in the fully connected dense layer. The output layer applied sigmoid as an activation function return from the model to be in the range from 0 to 1. (**E**) Diagnosis. The trained model diagnoses the subject as either early NTG (≥ 0.5) or GS (< 0.5). GS: glaucoma suspect; NTG: normal-tension glaucoma.

Recently, Bruch’s membrane opening-minimum rim width (BMO-MRW) has been presented in the estimation of discs, as a new parameter^16–20^. BMO-MRW is measured as the shortest length between the inner opening of the BMO and the internal limiting membrane (Fig. 1A). BMO-MRW offers more correct assessment of the NRR than traditional ophthalmic examination^16–18,21^. Recent studies have also reported that BMO-MRW showed better diagnostic performance of glaucoma than preexisting NRR parameters^22–24^. BMO-MRW has been demonstrated to have a stronger correlation with the VF than other disc parameters or RNFL^24,25^. Few previous studies using deep-learning investigated BMO-MRW for glaucoma diagnosis. One study by Park et al.^12^ reported the diagnostic performance of combined BMO-MRW and RNFL using a neural network for glaucoma but early glaucoma was only partly included and they also did not consider the RNFL color code classification.

Normal-tension glaucoma (NTG) is more prevalent in Asians than other races and NTG accounts for the majority (mean of 76.3%) of open-angle glaucoma in Asians^26^. However, previous deep-learning studies classifying glaucoma and normal subjects rarely included NTG and studies solely on NTG are difficult to find.

In this retrospective cross-sectional study, we aimed to classify early NTG and GS using OCT imaging-based parameters including the new parameter, BMO-MRW, and peripapillary RNFL, along with the color classification of RNFL, based on a new model of deep-learning. We investigated the diagnostic performance of all combined three parameters along with each parameter alone regarding global and six Garway-Heath sectors using deep learning method. We aimed to assess the clinical usefulness of the new parameter, BMO-MRW, in combination with the conventional parameter, RNFL, in the diagnosis of early NTG. We employed a deep-learning model to integrate all available data from the OCT images, which may be difficult for general physicians.

## Results

### Baseline characteristics of the datasets

A total of 397 eyes (397 subjects) with either GS (229 subjects) or early NTG (168 subjects) were included in the final analysis. The mean age of the subjects with GS was 47.68 ± 12.99 years, which was significantly younger than that of the subjects with early NTG at 55.95 ± 11.72 years (*p* < 0.001). The baseline IOP was not significantly different between GS and early NTG, which was 14.94 ± 2.60 mmHg and 14.76 ± 2.60 mmHg, respectively.

**Figure 2:**
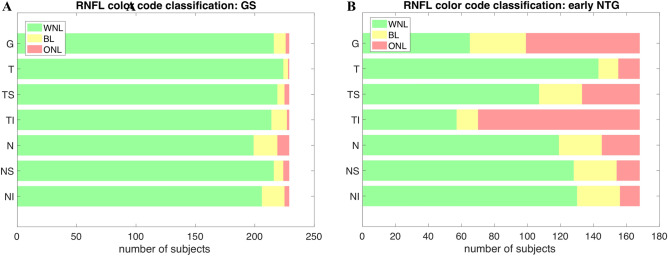
Proportion of WNL/BL/ONL in the RNFL color code classification of subjects (GS or early NTG). (**A**) RNFL classification (G, T, TS, TI, N, NS, and NI) of GS (n = 229) distributed by WNL/BL/ONL. The majority of subjects (over 200 out of 226) with GS were classified as WNL for all regions of RNFL classifications. (**B**) RNFL classification of early NTG (n = 168) distributed by WNL/BL/ONL. The proportion of ONL in RNFL G and RNFL TI was higher than that of WNL. RNFL, retinal nerve fiber layer. RNFL classification: WNL, within normal limits. BL, borderline. ONL, outside normal limits. RNFL: G, global. T, temporal. TS, superotemporal; NS, superonasal; N, nasal; NI, inferonasal; TI, inferotemporal.

The mean deviation (MD) for GS, − 0.80 ± 2.17 dB, was significantly higher than that for early NTG at − 2.86 ± 2.53 dB (*p* < 0.001). The pattern standard deviation (PSD) and visual field index (VFI) were also significantly different between GS and early NTG (all *p* < 0.001). The central corneal thickness (CCT) and spherical equivalent were significantly different between GS and NTG, which showed more myopic and thicker corneas for GS than early NTG (all *p* < 0.045). The detailed baseline characteristics are shown in Table 1. Pre-perimetric glaucoma was included in 33 subjects (19.60%) in the early NTG group. Table 2 shows the values of BMO-MRW and RNFL of the subjects with GS and early NTG. The global BMO-MRW values were significantly different between GS and early NTG (263.30 ± 41.57 um and 206.77 ± 43.96 um, respectively, *p* < 0.001). Six sectors of the BMO-MRW (T, TS, TI, N, NS, and NI) values were also significantly different between GS and NTG, as shown in Table 2. The mean global RNFL thickness of the patients with GS was 102.10 ± 10.05 µm, and that of the early NTG patients was 82.71 ± 13.14 µm (*p* < 0.001). The RNFL thickness of six sectors (T, TS, TI, N, NS, and NI) was also significantly different between the subjects with GS and early NTG (*p* < 0.001). Regarding global RNFL color code classifications, 94.3%, 4.4%, and 1.3% of the GS patients and 38.7%, 20.2%, and 41.4% of the early NTG patients showed within normal limits (WNL), borderline (BL), and outside normal limits (ONL), respectively. The majority of subjects with GS (over 200 out of 226 subjects) were classified as WNL for all regions of RNFL classifications as shown in Fig. 2A. On the other hand, Fig. 2B showed that for early NTG, ONL was accounted for higher proportion than that of WNL and BL in RNFL G and RNFL TI. Six sectors (T, TS, TI, N, NS, and NI) of the RNFL color code classification for GS and early NTG also had significantly different proportions of WNL, BL, and ONL (*p* < 0.05).

**Table 1:** Baseline characteristics of glaucoma suspect and early NTG subjects.

Characteristics	GS	Early NTG	*p* value
Number of subjects	229 eyes (229 subjects)	168 eyes (168 subjects)	
Mean age (years)	47.68 ± 12.99	55.95 ± 11.72	**< .001**
Female gender (%)	123 (54.18%)	75 (44.1%)	0.068
Family history of glaucoma (%)	13 (5.73%)	17 (9.94%)	0.072
Spherical equivalent (D)	− 1.97 ± 2.93	− 1.47 ± 2.68	**0.045**
CCT (um)	547.13 ± 39.07	537.30 ± 56.50	**0.014**
Baseline IOP (mmHg)	14.94 ± 2.60	14.76 ± 2.60	0.557
VFI (%)	98.51 ± 4.14	93.25 ± 6.89	**< .001**
MD (dB)	− 0.80 ± 2.17	− 2.86 ± 2.53	**< .001**
PSD (dB)	2.16 ± 1.39	4.69 ± 3.11	**< .001**

NTG, normal tension glaucoma; GS, glaucoma suspect; OCT, optical coherence tomography; D, diopters; CCT, central corneal thickness; IOP, intraocular pressure; VFI, visual field index; MD, mean deviation; PSD, pattern standard deviation. Results comparison with GS and early NTG are done with Wilcoxon signed-rank test, bold font indicates significant *p* values (*p* < 0.05).

**Table 2:** Bruch’s membrane opening-minimum rim width and retinal nerve fiber layer of the included subjects.

Characteristics	Values		*p* value
	GS (n = 229)	Early NTG (n = 168)	
**BMO-MRW**			
BMO-fovea angle°	− 5.43 ± 3.19	− 5.70 ± 3.19	.097
BMO area (mm^2^)	2.45 ± 0.53	2.30 ± 0.55	**.020**
BMO-MRW G (um)	263.30 ± 41.57	206.77 ± 43.96	**< .001**
BMO-MRW T	192.97 ± 41.16	159.54 ± 38.02	**< .001**
BMO-MRW TS	267.89 ± 43.06	206.40 ± 60.51	**< .001**
BMO-MRW TI	295.75 ± 53.04	196.31 ± 65.92	**< .001**
BMO-MRW N	275.96 ± 55.72	228.40 ± 58.06	**< .001**
BMO-MRW NS	291.02 ± 57.55	234.69 ± 62.61	**< .001**
BMO-MRW NI	321.98 ± 54.94	236.53 ± 63.80	**< .001**
**RNFL thickness**			
RFNL G (µm)	102.10 ± 10.05	82.71 ± 13.14	**< .001**
RFNL T	78.63 ± 12.24	66.51 ± 13.17	**< .001**
RFNL TS	142.62 ± 21.31	112.09 ± 29.72	**< .001**
RFNL TI	156.01 ± 19.66	101.73 ± 35.21	**< .001**
RFNL N	77.58 ± 15.63	68.21 ± 14.57	**< .001**
RFNL NS	114.78 ± 25.65	100.83 ± 27.29	**< .001**
RFNL NI	113.61 ± 23.78	91.91 ± 20.14	**< .001**
**RFNL classification (WNL/BL/ONL)**			
RFNL G (µm)	WNL (94.3%), BL (4.4%), ONL (1.3%)	WNL (38.7%), BL (20.2%), ONL (41.4%)	**< .001**
RFNL T	WNL (97.8%), BL (1.7%), ONL (0.4%)	WNL (85.1%), BL (7.1%), ONL (7.7%)	**.049**
RFNL TS	WNL (95.6%), BL (2.6%), ONL (1.7%)	WNL (63.7%), BL (15.5%), ONL (20.8%)	**< .001**
RFNL TI	WNL (93.4%), BL (5.7%), ONL (0.9%)	WNL (33.9%), BL (7.7%), ONL (58.3%)	**< .001**
RFNL N	WNL (86.9%), BL (8.7%), ONL (4.4%)	WNL (70.8%), BL (15.5%), ONL (13.7%)	**< .001**
RFNL NS	WNL (94.3%), BL (3.5%), ONL (2.2%)	WNL (76.2%), BL (15.5%), ONL (8.3%)	**< .001**
RFNL NI	WNL (90.0%), BL (8.3%), ONL (1.7%)	WNL (77.4%), BL (15.5%), ONL (7.1%)	**.002**

BMO-MRW, Bruch’s membrane opening-minimum rim width. RNFL, retinal nerve fiber layer: G, global. T, temporal. TS, superotemporal. NS, superonasal. N, nasal. NI, inferonasal. TI, inferotemporal. Statistical analysis between GS and early NTG for BMO-MRW and RNFL thickness was done by Wilcoxon signed-rank test, Bold font indicates significant *p* values (*p* < 0.05); RNFL classification: WNL, within normal limits. BL, borderline. ONL, outside normal limits. Statistical analysis between GS and early NTG was done by Mann–Whitney U Test for RNFL classification. Bold font indicates significant *p* values (*p* < 0.05).

### Correlation analysis of OCT-based parameters for classifying GS and NTG

**Figure 3:**
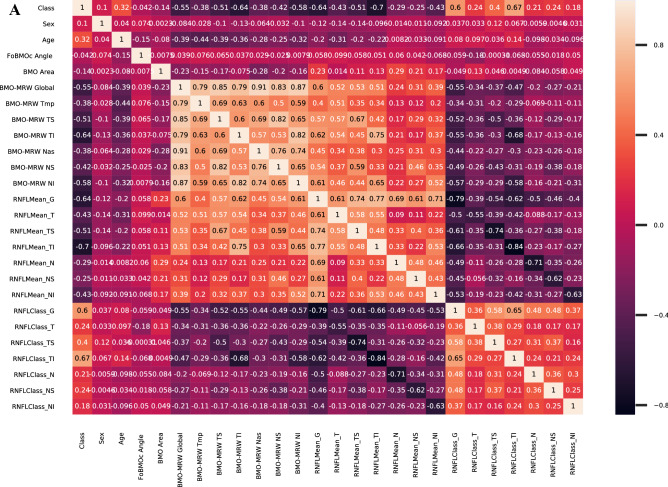

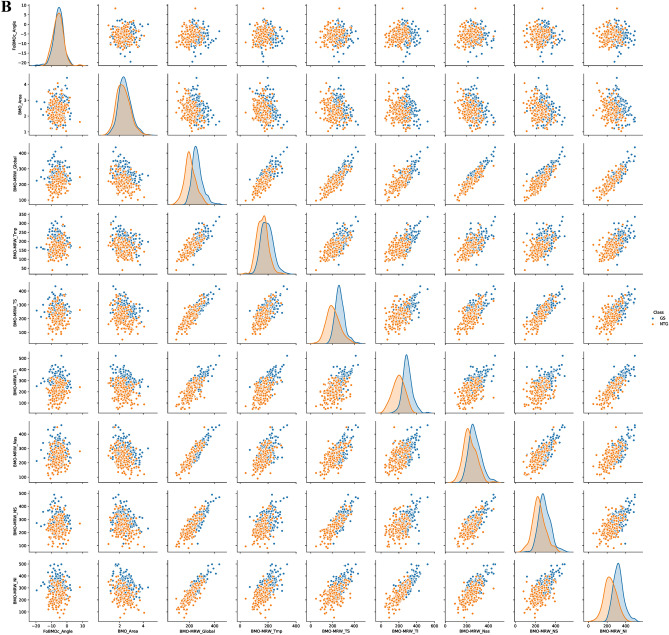
Correlation analysis of OCT-based parameters. (**A**) Heatmap of pairwise Pearson’s correlations between parameters (gender, age, BMO-MRW, RNFL) including class (either GS or early NTG). The negative correlations are in purple and the positive correlations in orange. The numbers are correlation coefficients in the range from − 1 to 1. RNFL TI, RNFL global, BMO-RMW TI, RNFL classification TI, and RNFL classification global showed − 0.7, − 0.64, − 0.64, 0.67, 0.6 Pearson’s coefficients with the class (GS = 0, early NTG = 1), respectively. (**B**) Scatterplots of the joint relationship and univariate distribution of the BMO-MRW parameters. The different categorical classifications are shown in blue (GS) and orange (early NTG). Subjects with GS and early NTG were clearly discriminated through sub-parameters in the order of the BMO-MRW TI, TS, and global. BMO-MRW, Bruch’s membrane opening-minimum rim width. RNFL, retinal nerve fiber layer: G, global. T, temporal. TS, superotemporal. NS, superonasal. N, nasal. NI, inferonasal. TI, inferotemporal.

We established a statistical relationship of pairwise parameters, which included the class (GS = 0, early NTG = 1), gender, the OCT-based parameters of BMO-fovea angle, BMO Area, BMO-MRW (global, T, TS, TI, N, NS, and NI), RNFL (global, T, TS, TI, N, NS, and NI), and the RNFL color code classification (global, T, TS, TI, N, NS, and NI). Figure 3A shows the heatmap of Pearson’s correlations with pairwise sub-parameters in the range from -1 to 1. RNFL TI, RNFL global, BMO-RMW TI, RNFL classification TI, and RNFL classification global showed − 0.7, − 0.64, − 0.64, 0.67, and 0.6 as Pearson’s coefficients with the class (GS = 0, early NTG = 1), respectively. This implies that RNFL TI, RNFL global, BMO-RMW TI, RNFL classification TI, and RNFL classification global were significant factors correlated with discriminating the class of either GS or early NTG. In addition, RNFL global has positive correlations with the RNFL TI (Pearson’s coefficient = 0.77), RNFL TS (Pearson’s coefficient = 0.74), and RNFL NI (Pearson’s coefficient = 0.71) values. Figure 3B shows scatterplots for the joint relationship and the univariate distribution of BMO-MRW parameters with different categorical classifications in blue (GS) and orange (early NTG), respectively. BMO-MRW TI, TS, and global discriminated the classes of GS (blue) and early NTG (orange). The subjects with GS and early NTG were clearly discriminated in the order of BMO-MRW TI, TS, and global sub-parameters, as shown in Fig. 3B. There were no significant differences between the two classes in terms of age, gender, and BMO-fovea angle. BMO-MRW global, IT, TS NI, and RNFL thickness global, TI, and TS were significant key parameters in distinguishing between GS and early NTG.

### Diagnostic performance of discriminating early NTG and GS

**Figure 4:**
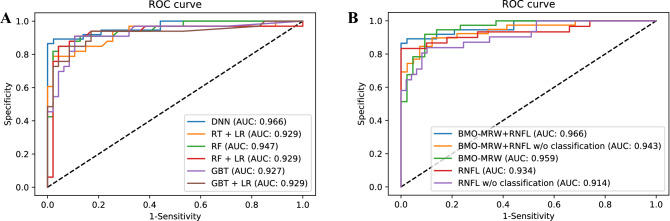
The area under the curve (AUC) of receiver operation characteristic curve (ROC) comparison. (**A**) evaluates ROC/AUC for the performance of machine learning models in testing dataset. Note that the DNN model provides the highest AUC [0.966 (95% CI 0.929–1.000)] in classifying either GS or early NTG, and other machine learning models showed AUCs in the range of 0.927–0.947. **(B)** evaluates ROC/AUC of DNN model with different set of OCT based parameters to evaluate the performance of diagnosis in testing dataset**.** Using all three parameters of BMO-MRW and RNFL combined with RNFL color code classification [AUC 0.966 (95% CI 0.929–1.000)] demonstrated the highest diagnostic performance than a single parameter or combination of just two parameters. As a single parameter, BMO-MRW [AUC 0.959 (95% CI 0.921–0.997)] demonstrated higher diagnostic performance than RNFL alone [AUC 0.914 (95% CI 0.850–0.979)] or even RNFL with color code classification [AUC 0.934 (95% CI 0.868–1.000)]. DNN: deep neural network. RT + LR: random trees with logistic regression. RF: random forest. RF: random forest with logistic regression. GBT: gradient boosted trees. GBT + LR: gradient boosted trees with logistic regression. BMO-MRW: Bruch’s membrane opening-minimum rim width. RNFL: retinal nerve fiber layer. BMO-MRW + RNFL: BMO-MRW and RNFL thickness with RNFL color code classification. BMO-MRW + RNFL w/o classification: BMO-MRW and RNFL thickness without RNFL color code classification. BMO-MRW: BMO-MRW only. RNFL: RNFL only with RNFL color code classification. RNFL w/o classification: RNFL thickness without RNFL color code classification.

We evaluated the performance of machine-learning models, as well as DNN, in the testing dataset. In the ROC curves, the DNN model provided the highest AUC of 0.966 (95% confidence interval [CI] 0.929–1.000) in classifying either GS or early NTG. The AUCs of logistic regression with a random tree (RT + LR), random forest (RF), random forest with logistic regression (RF + LR), gradient-boosting tree (GBT), gradient-boosting tree with logistic regression (GBT + LR) were 0.929 (95% CI 0.866–0.992), 0.947 (95% CI 0.898–0.996), 0.929 (95% CI 0.857–1.000), 0.927 (95% CI 0.864–0.990), and 0.929 (95% CI 0.857–0.993), respectively. The ROC curves for each model are shown in Fig. 4A.

The DNN model for the diagnosis, considering all OCT-based parameters with BMO-MRW, RNFL thickness, and RNFL color code classification provided the AUC of 0.966 (95% CI 0.929–1.000) and the accuracy of 96.2%. For the DNN model with BMO-MRW and RNFL thickness without RNFL color classification, the AUC was 0.943 (95% CI 0.894–0.992) with the accuracy of 87.5%. The AUC was 0.959 (95% CI 0.921–0.997) for the DNN model considering only BMO-MRW and the accuracy was 86.3%. For the performance of the DNN model with RNFL thickness and RNFL color code classification, the AUC was 0.934 (95% CI 0.868–1.000) with the accuracy of 91.2%. For the DNN model considering the RNFL thickness only without color code classification, the AUC was the lowest as 0.914 (95% CI 0.850–0.979) and the accuracy was 85.0%. The ROC curves of DNN with different parameters in the testing dataset are shown in Fig. 4B. The AUC of the DNN model with all OCT-based parameters (95% CI 0.929–1.000) was the highest compared to a single parameter or the combination of just two parameters (0.914 – 0.959). The accuracy was also the highest (96.2%) when all OCT-based parameters were considered for analysis. In addition, as a single parameter, the AUC of the DNN model considering the BMO-MRW only (0.959 [95% CI 0.921–0.997]) was higher than that of the model with only RNFL thickness without RNFL color classification (0.914 [95% CI 0.850–0.979]) or with RNFL thickness and RNFL color classification (0.934 [95% CI 0.868–1.000]).

## Discussion

To the best of our knowledge, the current study was the first to investigate the diagnostic performance of the new parameter, BMO-MRW, along with RNFL and its color code classification, in discriminating early NTG and GS using a deep-learning model. We found that the diagnostic performance of the DNN model was outstanding along with the other machine-learning models in classifying either early NTG or GS. The DNN model provided the highest AUC (0.966) compared to other machine-learning models (0.927–0.947). We also found that using all three parameters of BMO-MRW and RNFL combined with the RNFL color code classification demonstrated the highest diagnostic performance (0.966) compared to a single parameter or the combination of just two parameters. As a single parameter, BMO-MRW (0.959) demonstrated the higher diagnostic performance than that of RNFL alone (0.914) or RNFL with color code classification (0.934). Among the six Garway-Heath sectors, the inferotemporal sector was found to be useful in discriminating between early NTG and GS. The inferotemporal sector of the RNFL, BMO-MRW, and the color code classification of the RNFL all showed significant correlations with the class of early NTG or GS.

BMO-MRW from spectral-domain OCT has become increasingly available to clinicians and offers advantages compared to previous standard morphometric optic nerve head analysis confocal scanning laser tomographic measurements^22–24^. BMO-MRW provides a geometrically more accurate assessment of the NRR than preexisting ophthalmic examination^16–18,21^. It has been reported that BMO-MRW is advantageous to accurately reflect the amount of neural tissue from the optic nerve^27^. In our previous study, we reported a discrepancy between BMO-MRW and the RNFL color code classification^7^. We found that, in cases of large disc and myopia, for which glaucoma suspects are frequently referred, BMO-MRW may show a normal classification, whereas RNFL shows an abnormal classification. In such cases, BMO-MRW may suggest normal findings when the RNFL color code classification shows false-positive findings, which may lead to confusion in clinicians in the diagnosis of early-stage glaucoma. However, a consensus on the criteria discriminating abnormal from normal BMO-MRW and RNFL measurements is lacking. Integrating the assessment of BMO-MRW and RNFL is necessary for the better diagnosis of early glaucoma based on these findings. Nevertheless, the integration of these two different parameters is neither simple nor easy for human beings, including general physicians other than glaucoma specialists. This is where the recent technology of artificial intelligence can help. It has been reported that machine-learning classifiers can be beneficial in clinical practice and efficiently improve glaucoma diagnosis for general ophthalmologists in the primary eye care setting when there is no glaucoma specialist available^28^. The DNN model can provide prompt diagnostic results in the clinics after the input of ophthalmic examination data, not requiring a few days of analysis. Of course, the decision to treat glaucoma is up to the physician. However, the DNN model can suggest a preliminary diagnosis for reference^29^. The DNN diagnostic model is more economical and clinically easy to access than other imaging-based CNN diagnostic programs that take days and require expensive equipment, such as workstations with graphics processing units (GPUs).

To our knowledge, the color code classification of RNFL has not been evaluated in the deep-learning model before. In our study, we included the RNFL color code classification as one of the three parameters. The diagnostic performance of RNFL alone (AUC 0.914) was much lower than that of RNFL with its color code classification (AUC 0.943) or even BMO-MRW alone (AUC 0.959). The sensitivity of RNFL thickness defined from diagnostic color code classification report was compared at higher specificities (between 98.7 and 100%)^29^. High specificities were noticed in the color code classification analysis since the first and the fifth percentile of the normative RNFL thickness data were employed to determine the RNFL thickness abnormalities^29^. Therefore, the RNFL color code classification is important in the diagnosis of early glaucoma and it should be considered together with RNFL thickness for better diagnostic performance. The color code classification of BMO-MRW has not been considered in the present study, because BMO-MRW alone without color code classification was good enough to show higher diagnostic performance compared to RNFL alone or RNFL with color code classification. Moreover, color code classification for RNFL is a general diagnostic aid already widely used for clinicians. Compared to BMO-MRW, which is a relatively a new parameter, adding color code classification of RNFL would more benefit the clinical practice in glaucoma diagnosis.

It is more challenging to discriminate early stage of glaucoma from glaucoma suspect or normal subjects than advanced stage of glaucoma^9–11^. A previous study by Park et al.^12^ that investigated BMO-MRW and RNFL for glaucoma diagnosis only included 38 subjects with early glaucoma for training, and pre-perimetric glaucoma was excluded. Moreover, the diagnostic performance of the neural network by AUROC including both parameters was only 0.896 for early glaucoma. Compared to this previous study, our study included entirely early NTG and the AUROC was much higher, at 0.966 (95% CI 0.929–1.000), when BMO-MRW, RNFL, and its color code classification were considered. One more point is that we included an even earlier stage of glaucoma, pre-perimetric glaucoma. Thirty-three patients (19.60%) with pre-perimetric glaucoma in the early NTG group were included in our study. The diagnostic outcome of our DNN model for the very early stage of glaucoma included in our study was remarkable compared to the previous study. In another study that used hybrid deep-learning to distinguish healthy suspects with early glaucoma, wide-field OCT scans were used^15^. The accuracy ranged from 63.7 to 93.1%, depending upon the input map. The accuracy varied widely in their study and the accuracy of our DNN model with all parameters was 96.2%. The AUROC of all combined OCT images was 0.95 in their study^15^. The diagnostic performance of our DNN model was comparable to a previous study that evaluated the early stage of glaucoma using OCT images. Another recent study using deep-learning and transfer learning to diagnose early-onset glaucoma (MD of > − 5 dB) from macular OCT images reported an AUROC of 0.937^30^, which was less than that of our study (0.966). However, it is not clear that pre-perimetric glaucoma patients were included in these previous studies^15,30^ as it was not specifically described.

In correlation analysis of the OCT-based parameters for classifying GS and NTG, the inferotemporal sector, followed by the superotemporal sector of BMO-MRW and RNFL, showed significant correlations among the six Garway-Heath sectors. The site of the initial glaucomatous injury is correlated with the structure of the lamina cribrosa. It is well-recognized that the key site of glaucomatous damage is the lamina cribrosa^31–34^. Initial damage appears more often at the inferior and superior portion of the axons^35^ because a larger solitary-pore area in the lamina cribrosa increases glaucoma susceptibility in the inferior area, followed by the superior optic disc area^36,37^. For this structural vulnerability, glaucomatous change occurs mainly in the inferotemporal sector, followed by the superotemporal sector at the beginning of the disease. Therefore, these sectors, especially the inferotemporal sector, is important in the diagnosis of early glaucoma and also offers better diagnostic ability than other sectors.

In the Asian population, NTG consists primarily (76.3%) of patients with open-angle glaucoma, as described in population-based glaucoma-prevalence studies in Asians^26^. Therefore, discriminating NTG from glaucoma suspect is important in clinical practice in Asians. It applies to Asian countries and also to other countries worldwide with a significant Asian population proportion. Since deep-learning models regarding data with NTG are rare, the present study may have a significant meaning by adding information for reference and future studies.

The present study had some limitations. First, possible limitations of the present study exist in its retrospective nature. We included only subjects who had had both BMO-MRW and RNFL scans and had a reliable quality of both structural tests. The effect of such subject selection on our results is not known. Second, it was a hospital-based design carried out at a referral university hospital of the province, and not a population-based study. The included subjects might not represent the entire normal population. Another limitation is that this study included only Korean patients. The results of our study, including NTG, may not apply to other ethnic groups or other types of glaucoma. Third, the relatively small sample size of this study should also be taken into consideration. However, nearly 400 subjects with early NTG and GS were included in this study and this number is not insufficient to train and test diagnostic performance to classify a single disease from single device data. Lastly, in this study, OCT image-based analysis was conducted with numeric data extracted from the images, not direct image analysis using convolution neural networks (ConvNets). However, it is still meaningful that clinicians can obtain quick results and get aid in diagnosis by applying deep-learning models with free open-sources, compared to that most image analyses using ConvNets are yet less economical to achieve high accuracy. We may consider developing a program for the preliminary diagnosis from direct OCT-image analysis using ConvNets with the accuracy of its performance in the future study.

In conclusion, our DNN model showed high diagnostic performance considering all OCT-based parameters, including the new parameter of BMO-MRW along with RNFL and its color code classification, in discriminating early NTG and GS. BMO-MRW demonstrated higher diagnostic performance than other single parameters of RNFL or RNFL combined with its color code classification. The inferotemporal sector, among the six Garway-Heath sectors, was found to be most useful in diagnosing early NTG. Our DNN model may be beneficial in clinical practice in the diagnosis of early glaucoma, which is more challenging than that of advanced glaucoma. Since our DNN model provides a prompt output, it may be more useful in the primary eye care setting where glaucoma specialists are not available. A further multi-center study with a large patient number is required to draw more definitive conclusions.

## Materials and methods

### Ethics statement

This retrospective observational, cross-sectional study was performed according to the tenets of the Declaration of Helsinki. It was approved by the Institutional Review Board (IRB) of Gyeongsang National University Changwon Hospital, Gyeongsang National University School of Medicine. The requirement for informed consent was exempted from the IRB of Gyeongsang National University Changwon Hospital due to its retrospective nature.

### Subjects

Among 277 patients with GS and 285 patients with normal-tension glaucoma (NTG) who were evaluated between the period of February 2016 and April 2019 in a glaucoma clinic at Gyeongsang National University Changwon Hospital, a total of 397 eyes (397 subjects) with either GS (229 subjects) or early NTG (168 subjects), were included. Only those subjects with both reliable BMO-MRW and RNFL results and those who met the diagnosis criteria below were included. Assessment of early NTG or GS was evaluated by a single glaucoma specialist (H-k Cho) with consistent definition criteria.

A diagnosis of NTG was made when a patient with an IOP of ≤ 21 mmHg without treatment had findings of glaucomatous optic disc damage and corresponding VF defects, an open-angle observed by gonioscopic examination, and no underlying cause for optic disc damage aside from glaucoma^38^. Early NTG was determined as VF test results of mean deviation (MD) > − 6.0 dB. Pre-perimetric glaucoma was included in the present study to include the very early stage of glaucoma. Pre-perimetric glaucoma was defined as cases showing definite localized RNFL defects on red-free fundus photography with a confirmed corresponding RNFL defect in the OCT map of RNFL but within normal limits on Humphrey standard automated perimetry.

GS was defined as those being followed on the basis of suspicious clinical features but not conclusive for glaucoma, including suspicious optic disc or RNFL changes; significant systemic, ocular or family risk factors for glaucoma; or suspicious visual field results and intraocular pressure within normal limits (defined as < 21 mmHg on applanation tonometry). By definition, none of the glaucoma suspects were receiving treatment for glaucoma and treated ocular hypertensive patients were excluded^39^. Ocular hypertensive patients without treatment were also excluded. If both eyes met the inclusion criteria, only one eye was randomly selected for the study.

A normal classification shows green, whereas an abnormal classification shows yellow (borderline) or red (outside normal limits) on Spectralis spectral-domain optical coherence tomography (Glaucoma Module Premium Edition, Heidelberg Engineering, Germany). All subjects underwent standard ophthalmic examinations including Spectralis spectral-domain OCT and standard automated perimetry (HFA model 840; Humphrey Instruments Inc., San Leandro, CA, USA).

The exclusion criteria are as follows: poor image scans due to eyelid blinking or bad fixation, history of any intraocular surgery except for uneventful phacoemulsification, history of optic neuropathies other than glaucoma or an acute angle-closure crisis that could influence the thickness of the RNFL or BMO-MRW (e.g., optic neuritis, acute ischemic optic neuritis), and retinal disease accompanied by retinal swelling or edema and subsequent RNFL or BMO-MRW swelling. Subjects were not excluded by refractive error or axial length, and optic disc size for this study.

### Optical coherence tomography

OCT imaging of the spectral-domain was performed using the Glaucoma Module Premium Edition. Twenty-four radial B-scans were obtained for BMO-MRW. For peripapillary RNFL thickness, a scan circle of 3.5 mm in diameter among three scan circles (3.5, 4.1, and 4.7 mm in diameter) was used. Well-centered scans with correct retinal segmentation and quality score > 20 were accepted. Data acquisition and OCT analyses were performed according to the individual eye-specific axis (FoBMO axis), the axis between the BMO center and the fovea. FoBMO axis could lead to more accurate sectoral analysis considering cyclotorsion of individual eyes and more accurate comparison with normative data than the conventional method.

### Perimetry

We used a Humphrey Field Analyzer (HFA model 840; Humphrey Instruments Inc.) for perimetry with a central 30-2 program of Swedish Interactive Threshold Algorithm standard strategy. A reliable VF test had to fulfill three criteria: fixation loss less than 20%; false-positive rate < 15%; and a false-negative rate < 15%.

### Data processing

The dataset consisted of 229 eyes out of 277 GS (GS group) and 168 eyes of 285 patients with early NTG (early NTG group). The OCT-based images of 397 patients from BMO-MRW and RNFL were converted into numeric values, as shown in Fig. 1A,B. Then, we generated input data with two different classes, GS and early NTG, and each class consisted of sub-parameters, such as age and gender, and three main quantified ocular parameters, BMO-MRW, RNFL thickness, and the RNFL color code classification as shown in Fig. 1C. The input data were randomly divided into a training set and a testing set using programming language Python version 3.6.8 (https://www.python.org/). Out of 297 eyes, 217 eyes were used to construct a training set (80%) and 80 eyes as a test set (20%).

### Deep neural network architecture

A deep neural network (DNN) is a supervised classifier that contains multiple layers between the input and output layers^40^. Deep-learning technologies have led to the development of neural network (NN) architectures that have been shown to be useful classification tasks^41^. To discriminate GS from early NTG, we proposed a deep neural network model, as shown in Fig. 1D. We trained and tested our model built on Keras (https://keras.io/), the open-source neural network API, written in Python running on TensorFlow (https://www.tensorflow.org/)^42^. Our model consisted of the input layer, three hidden layers, and the output layer. The input layer received data with 25 sub-parameters based on three main quantified ocular parameters: BMO-MRW, RNFL thickness, and the RNFL color code classification. The first and second hidden layer had 10 neurons with an activation function of a rectified linear unit (ReLU), and the third hidden layer had five neurons with a ReLU in the fully connected dense layer. The output layer applying a sigmoid function as an activation function returned to be in the range from 0 to 1, thus the model predicted the probability of glaucoma, as shown in Fig. 1E.

We considered additional approaches to diagnose glaucoma with other machine-learning models with (i) a DNN, (ii) logistic regression with a random tree (RT + LR), (iii) a random forest (RF), (iv) a random forest with logistic regression (RF + LR), (v) a gradient-boosting tree (GBT), and (vi) a gradient-boosting tree with logistic regression (GBT + LR). We also evaluated additional approaches with various combinations of parameters to diagnose glaucoma: (i) BMO-MRW and RNFL thickness with RNFL color code classification, (ii) BMO-MRW and RNFL thickness without RNFL color code classification, (iii) BMO-MRW only, (iv) RNFL thickness with RNFL color code classification only, and (v) only RNFL thickness without RNFL color code classification.

### Statistical analysis

The demographic data were compared between the two groups of GS and early NTG using the Wilcoxon-signed rank test for continuous and categorical variables. Statistical significance was considered for *p-*values less than 0.05. We also evaluated Pearson’s correlation coefficients, which is a measure of the linear correlation of pairwise sub-parameters, including the class of either GS or early NTG. It has a value between − 1 and 1, where 1 is a total positive linear correlation, 0 is no correlation with each other, and − 1 is a total negative linear correlation between the two sub-parameters. To evaluate the classification performance of the deep-learning algorithm, the area under the curve (AUC) of the receiver operating characteristic curve (ROC), sensitivity, specificity, f1 score, and accuracy were utilized. All statistical analyses were performed using programming language Python version 3.6.8 (https://www.python.org/) and SPSS software version 24.0 (https://www.ibm.com/analytics/spss-statistics-software).
